# Retrospective Evaluation of Peri-Implant Maintenance in Patients With Implant-Supported Fixed Prostheses

**DOI:** 10.1155/ijod/9920951

**Published:** 2025-10-16

**Authors:** Poyan Maghsoudi, Cees Valkenburg, Lotte Pull ter Gunne, Fridus (G. A. ) van der Weijden

**Affiliations:** ^1^Department of Periodontology, Academic Center for Dentistry Amsterdam (ACTA), A Joint Venture Between the Faculty of Dentistry of the University of Amsterdam and Vrije Universiteit Amsterdam, Amsterdam, Netherlands; ^2^Freelance Statistician, Netherlands; ^3^Clinic for Implantology Utrecht, Utrecht, Netherlands

**Keywords:** bleeding on probing, emergence angle, maintenance care, pocket probing depth, two-stage implants

## Abstract

**Background:**

Dental implants have emerged as a dependable solution for replacing missing teeth, boasting high survival rates. Nonetheless, implant placement marks only the initiation of a lifelong maintenance protocol. Ensuring the long-term success of dental implants hinges on the essential preservation of peri-implant tissue health and is dependent on patient compliance, which is considered a critical determinant in supportive care.

**Objective:**

The purpose of this study is to retrospectively analyze peri-implant mucosal aspects from the time of baseline assessment to a follow-up period of 3.5–4.5 years.

**Material and methods:**

This retrospective analysis included follow-ups of 68 patients who received one or multiple dental implants between 2011 and 2016. Baseline assessment took place around 8 weeks after placement of the final restoration to assess the peri-implant condition clinically and radiographically. Peri-implant bleeding on probing (PiBOP), peri-implant probing pocket depth (PiPPD), and gingival recession were recorded during every visit.

**Results:**

Implant-level analysis of PiBOP showed an increase from 31% at baseline to 48% at the follow-up appointment (*p* < 0.001). The average PiPPD at implant level was 3.2 mm at baseline and increased to 3.5 mm (*p* < 0.001). Male patients presented with significantly higher PiPPD values compared to female patients (implant level: *p*=0.017, patient level: *p*=0.039). There was no apparent difference in PiPPD between the group with a restoration with an emergence angle <30° or ≥30° (*p*=0.912).

**Conclusion:**

Peri-implant conditions remained fairly stable after the follow-up period. More effort needs to be made to improve the adherence of patients to peri-implant maintenance care.

**Summary**

• What is known on the topic: The preservation of peri-implant tissue health is of importance for ensuring the long-term success of dental implants in clinical practice.
• This study adds: Of the total group of patients that had received implants only one-third adhered to the annual maintenance visits and supportive care. Results indicate that after a follow-up period of 3.5–4.5 years, patients with fixed implant-supported restorations exhibited a relatively stable peri-implant condition. There appears to be a necessity for increased efforts to enhance patient adherence to peri-implant maintenance care.

## 1. Introduction

Dental implants are currently a well-established treatment option in the replacement and restoration of missing teeth with predictable outcomes and high survival rates [1]. With increasing survival rates, peri-implant tissue health has become of greater importance. Achieving and preserving peri-implant soft tissue health is crucial in achieving long-term survival [2]. The most recent classification for peri-implant health in day-to-day clinical practice is absence of clinical signs of inflammation, absence of bleeding/suppuration on gentle probing, no increase in probing depth compared to previous examinations, and no bone loss [3].

The peri-implant tissue condition can be assessed based on several clinical parameters. Visual inspection and palpation are clinical methods used to detect inflammation at an implant site. Peri-implant probing is the more reliable and important diagnostic tool in longitudinal monitoring of peri-implant bleeding on probing (PiBOP) and peri-implant probing pocket depth (PiPPD) and should be performed using a light force (0.2–0.25 N) [4]. The absence of bleeding on probing is a reliable predictor for periodontal stability and is, therefore, a valuable tool in monitoring peri-implant tissue health and for diagnosis of mucosal inflammation [5, 6]. In addition, it is advised and justified to make radiographs at predetermined intervals to obtain essential diagnostic information regarding peri-implant marginal bone levels [7, 8]. All information gathered at a baseline assessment provides information necessary to gain insight into the development of the peri-implant tissue health in subsequent examinations. It is advised to perform the baseline assessment around 8 weeks after restoration placement and should preferably include the following parameters: PiPPD, PiBOP, exudate, implant mobility, cleanability, and occlusion supplemented with clinical photographs and radiographs [9]. While these clinical parameters remain standard diagnostic tools, we acknowledge that emerging methodologies, such as the sampling of peri-implant sulcus fluid for pro-inflammatory biomarkers, may allow for earlier detection of disease onset [10, 11]. However, such techniques are not yet routinely used in general practice and were therefore not included in the current study.

Besides regular clinical and radiographic examinations, maintenance/supportive care of the peri-implant tissues needs to be addressed [12]. Supportive treatment during a maintenance period can potentially improve the survival rate, prevent peri-implant mucositis, and peri-implantitis and in doing so improve overall peri-implant health [13]. A supportive maintenance program has been proven as essential for the long-term success of implants [10, 12]. Several factors such as poor oral hygiene, a history of periodontitis, lack of compliance with supportive periodontal therapy and smoking have been identified as potential risk indicators for peri-implant disease [14–16]. Restoration-related factors may also act as a risk indicator in the development of peri-implantitis. These findings are supported by recent studies showing that a restoration emergence angle ≥30° on bone-level implants serves as a significant risk indicator for peri-implant disease [17, 18].

This retrospectively analysis evaluated peri-implant mucosal aspects from the time of the baseline assessment to a follow-up period of 3.5–4.5 years in a cohort of patients who received fixed implant-supported restorations. During this period clinical assessments of the peri-implant tissue health were performed, and supportive care was provided on a regular basis to the patient.

## 2. Material and Methods

This retrospective analysis is reported in accordance with the Strengthening the Reporting of Observational Studies in Epidemiology guidelines for reporting observational studies (STROBE) [19]. The protocol was reviewed and approved by the Institutional Review Board of the Academic Centre for Dentistry Amsterdam under protocol number 2021-25339.

### 2.1. Population

This retrospective analysis included follow-ups of patients who received one or multiple dental implants between 2011 and 2016 at a specialist clinic in Utrecht, the Netherlands. This private clinic is confined to only implant dentistry. Either a 3.3 mm narrow-diameter or 4.1 mm regular-diameter bone-level Straumann implant (Institute Straumann AG, Basel, Switzerland) was used for partially edentulous patients. Patients were considered eligible for analysis if they met the following criteria:

• In good general health
• Age: ≥18 years
• Baseline assessment available
• Emergence angle of the abutment measurable on the radiograph
• Periodontally healthy (pocket ≤5 mm) [20]
• An implant-supported crown or a fixed partial denture (bridge) as a restoration.

Patients had to follow the sequence of appointments as stated in the 5th ITI Consensus Statements [21]. Furthermore, data from a monitoring visit had to be available after 3.5–4.5 years. Patients were excluded if there had been missing data from the baseline assessment and follow-up appointment after 3.5–4.5 years or if they received implant-supported removable dentures. Patients with well-controlled diabetes or taking medication, such as anticoagulants or contraceptive pills, were included in this analysis as well as patients under periodontal maintenance care.

### 2.2. Surgical and Prosthetic Treatment

The way the surgical and prosthetic treatments are performed are thoroughly described in a previous retrospective analysis on marginal bone level changes around two-stage implants [22]. In brief, two experienced implant dentists, accredited by the Dutch Society of Oral Implantology (NVOI), performed the two-stage surgical procedures under sterile conditions according to the manufacturer's instructions [23]. Two days before the implant surgery all patients were provided an antimicrobial prophylaxis (amoxicillin, 375 mg, three times daily) to reduce the risk of implant failure up to 3 days afterwards [24]. If patients reported an allergy to penicillin, clindamycin was prescribed as an alternative. To achieve successful coverage, the operator used a crestal incision technique in combination with a vertical distal and mesial incision and horizontal periosteal-releasing incision [25]. To increase the bone width, a bovine grafting material (Bio-Oss, Geistlich Pharma, AG, Wolhusen, Switzerland) was placed in all cases on the buccal site of the bone which was then covered by a resorbable membrane (ACE RCM6, ACE Surgical Supply Co., Brockton, MA, USA). At the second stage surgery, which took place three to 6 months after the implant placement, the operator exposed the implant and placed a healing abutment high enough to perforate the peri-implant mucosa. After 6 weeks of healing of the mucosa prosthetic procedures were started. Patients received either a screw-retained crown or fixed partial denture (bridge) restoration.

### 2.3. Clinical and Radiographical Assessments

Clinical assessments were performed following abovementioned recommendations on peri-implant maintenance care [21]. This implied a baseline assessment around 8 weeks after placement of the final restoration to assess the peri-implant condition clinically and radiographically. PiBOP, PiPPD, and gingival recession were recorded during every visit using a pressure-sensitive probe (Click-Probe, Kerr Hawe scale: 3–5–7–10 mm). The PiPPD was measured at six sites around the implant and each measurement was rounded off to the nearest millimeter. If the operator deemed it necessary, plaque and calculus were professionally removed during every maintenance visit with carbon fiber hand instruments and/or an air polisher (EMS Dental, Nyon, Switzerland) with erythritol prophylaxis powder (EMS Dental, Nyon, Switzerland). To achieve successful mechanical removal of bacterial plaque, all patients were provided individual oral hygiene instructions. The operator followed the same clinical measurements during the maintenance visits as at the baseline assessment.

**Figure 1:**
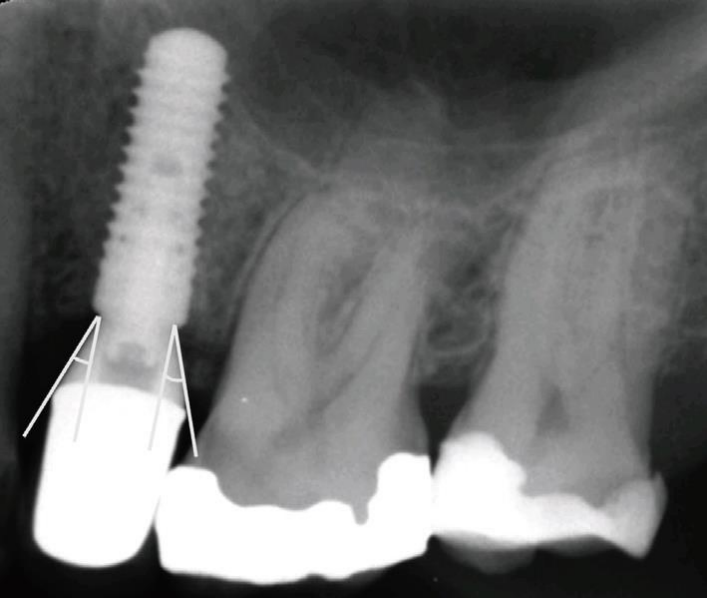
Radiograph showing measurements of the emergence angle relative to the longitudinal axis of the implant.

Furthermore, radiographs were taken at the baseline assessment using the long-cone paralleling technique with aiming devices (Dentsply Rinn XCP, Dentsply Sirona Benelux, Netherlands). The emergence angle was measured on the mesial and distal aspect of the implant, as presented in Figure 1. These measurements were performed by one and the same examiner (Poyan Maghsoudi) and were rounded off to the nearest degree. The baseline assessment served as a reference of the changes in peri-implant tissue health.

### 2.4. Statistical Analysis

Patient-related variables were collected in an EXCEL file which was aangleymized so that personal information pertaining to individual patients, both direct and indirect, were made untraceable. Both the implant as the patient served as the unit of analysis. If a patient had multiple implants, the related variables were averaged into patient level data. Quantitative data are presented as means with standard deviations or the number of cases with percentages. For statistical analysis of PiBOP and PiPPD, a paired-samples *T*-test was performed. To compare results between two subgroups an independent *T*-test was conducted. A multivariable linear regression was performed to determine the association between PiPPD at follow-up and PiPPD at baseline, mean emergence angle, smoking, gender, and age at implant placement. A one-way ANCOVA was conducted to test differences in PiPPD between groups on the number of maintenance visits controlling for baseline PiPPD. Cases with missing data (i.e., missing data from clinical examinations) were excluded from all analysis. SPSS version 27.0 (SPSS Inc., Chicago, IL, USA) was used for statistical analysis. *p*-Values <0.05 were set as the threshold value for statistical significance.

## 3. Results

**Figure 2:**
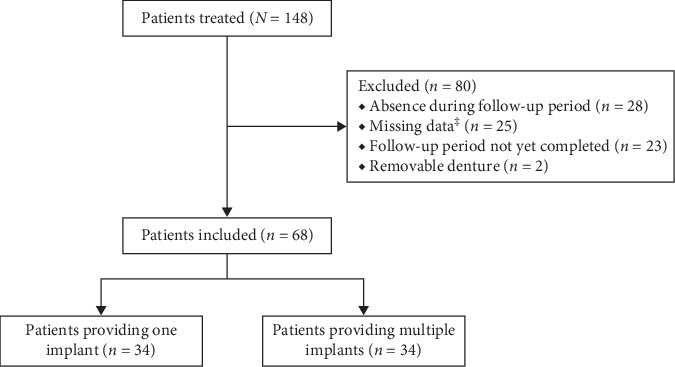
Flow chart of including patients with reasons to exclude. ^‡^Incomplete or missing evaluations at baseline assessment or follow-up visit, while the subject fulfilled the follow-up period of the study.

During the review period between 2011 and 2016, 148 patients had received implant placement therapy. Eighty patients (54%) were excluded for not meeting the inclusion criteria, thus, leading to 68 selected patients (46%; Figure 2). The most common reason for exclusion was absence during follow-up period (35%), followed by missing data (31%). Descriptive data of the included patients are presented in Table 1. The average age of the selected patients at implant placement was 57 years (range 33–74 years). Forty-two (62%) patients in this retrospective analysis were female and the average follow-up duration was 47 months. Forty-four (65%) patients had pockets equal or greater than 5 mm. Seventy-six (70%) implants were placed in the maxilla and the overall distribution in implant diameter was roughly equally distributed (3.3-mm: 48% vs 4.1-mm: 52%). Two-thirds (72) of the implants received a crown restoration, while the other third (36) of the implants served for implant-supported fixed restorations (bridge).

Analysis of PiBOP and PiPPD, both at implant and patient level, demonstrated a significant increase in PiBOP and PiPPD between the baseline assessment and follow-up appointment (Tables 2 and 3). Implant-level analysis of PiBOP showed an increase from 31% at baseline to 48% at the follow-up appointment (*p* < 0.001). A similar significant increase (19%) was found at patient-level analysis (*p* < 0.001). The average PiPPD at implant level was 3.2 mm at baseline and increased to 3.5 mm (*p* < 0.001) with a range from 2 to 6 mm. A comparable increase was observed at patient level (0.4 mm, *p* < 0.001).

The numerical increase of PiPPD in the narrow-diameter implant group (0.48 mm; *p* < 0.001) was found to be higher than the wide-diameter group (0.17 mm, *p*=0.097; Table 2). However, this difference between the two groups was found not to be statistically significant (*p*=0.074). Male patients presented with significantly higher PiPPD values compared to female patients (implant level: *p*=0.017; patient level: *p*=0.039). The difference in PiPPD between the baseline and follow-up measurements was approximately three times higher in male subjects (Tables 2 and 3). No significant differences were found between anterior vs. posterior and maxilla vs. mandibula (*p*=0.599, *p*=0.926, Table 2). There was no apparent difference in PiPPD between the group with a restoration with an emergence angle <30° or ≥30° (*p*=0.912; Table 2). Both showed a similar increase in PiPPD through time. No difference was found between patients with pockets ≥5 mm or <5 mm around their natural teeth (*p*=0.118; Table 3) nor between patients smoking and not smoking (*p*=0.536; Table 3).

A multivariable linear regression was conducted where the mean PiPPD at follow-up was the dependent variable (Table 4). These variables (Table 4) combined explained 23% of the variance in the PiPPD at follow-up (*R*^2^ = 0.23). The baseline PiPPD value and gender were found to be significantly associated with the PiPPD at follow-up. Male subjects had 38% higher values of PiPPD at follow-up compared to female subjects (*p* < 0.001).

## 4. Discussion

It has been acknowledged that in order to achieve long-term implant success regular maintenance care is of great importance [13, 26]. This retrospective analysis evaluated changes in PiPPD and PiBOP over a follow-up period of 3.5–4.5 years in a cohort of patients that received one or multiple implants. Of the total group of patients that had received implants only one-third adhered to the annual maintenance visits and supportive care. Results demonstrated a small, but significant increase in PiBOP and PiPPD in time. Both baseline PiPPD and gender were found to be significant predictors of an increase in PiPPD at follow-up. The analysis also investigated the emergence angle in relation to changes of PiPPD, but found no significant correlation. The difference in PiPPD (probing implant pocket probing depth) between the narrow-diameter implant group and the wide-diameter group was not found to be statistically significant. These results align with those of a 3-year split-mouth randomized clinical trial, which demonstrated no significant differences in terms of marginal bone loss, implant survival, and success rates between the two groups [27]. Furthermore, this study has enhanced our views concerning peri-implant maintenance care. Implant therapy should not be confined to only placement, but these patients should also be informed about the importance of regular recalls. A correct and strict maintenance protocol promotes peri-implant health, lowers the risk of implant failure, and in doing so heightens the long-term success rate [28].

### 4.1. PiBOP

The results show a significant increase in PiBOP, both at the implant and patient levels (Tables 2 and 3). Bleeding was evident at 48% of implant sites, which indicates a possible presence of inflamed peri-implant tissue. However, bleeding on probing does not necessarily have to indicate inflammation and may be caused by disrupting the epithelial attachment. PiBOP may be of poor diagnostic value [29]. If bleeding is observed, the profuseness and the occurrence of suppuration has been found to correlate with peri-implant bone loss, whereas minimal bleeding did not [30]. In addition, a probing force of 0.15 N was proven to represent the threshold pressure to avoid false positive PiBOP readings. Increasing the probing pressure by 0.1 N from 0.15 N resulted in 13.7% higher PiBOP values [31]. Therefore, PiBOP on its own is no true sign of inflammation and could be deceptive [30]. The probability of PiBOP is also associated with site-specific factors (i.e., position and PiPPD) and patient-related factors (i.e., gender) [31].

### 4.2. PiPPD

The results also show a significant increase in PiPPD, both at the implant and patient levels (Tables 2 and 3). PiPPD increased from 3.2 mm at baseline to 3.5 mm at follow-up (Table 2). Although the increase is statistically significant, the effect size is small and most likely not clinically relevant. It should be realized that PiPPD of healthy peri-implant tissues is not always <4 mm, as long-term research shows, but can vary between 4 and 6 mm [32]. The PiPPD and PiBOP findings of this retrospective analysis correlate well with the results of a 5-year follow-up study [33].

### 4.3. Clinical Signs Around Implants

It has been suggested that dental implants showing signs of inflammation, such as bleeding on probing and increased probing pocket depth, should be treated similarly to natural teeth affected by periodontitis. This recommendation assumes that periodontal indices, such as probing pocket depth and bleeding on probing, are reliable indicators of the condition of the peri-implant tissues, and that an increase in probing pocket depth overtime and the presence of bleeding on probing can accurately predict future bone loss and implant failure. However, this approach carries the risk of over-diagnosing peri-implant pathology, leading to unnecessary and possibly harmful treatment for patients [32]. Therefore, in order to monitor the peri-implant tissue health, as advised by a recent review, PiBOP assessments should be combined with changes in PiPPD and marginal bone levels in relation to a baseline assessment [34]. It is recommended to assess PiBOP not on a dichotomous scale (present/absent), but rather on scales based on profuseness [35] and/or time after probing which is an aspect to consider for future studies [32, 36]. Often, deeper probing pocket depths are found at implant sites inserted in partially edentulous ridges. The differences in the mucosal thickness can explain these findings [32]. Also PiPPD measurements are more sensitive to force variation compared with periodontal pocket probing [37, 38].

### 4.4. Gender

Male patients presented three times higher change in PiPPD increase throughout time compared to female patients (Tables 2 and 3). In addition, the regression analysis revealed that gender (male) is a significant predictor for increased PiPPD at follow-up (Table 4). The findings of this present analysis are consistent with a similar study that also observed more increase in PiPPD in male patients [39]. However, there seems to be some ambiguity regarding the association between gender and increased PiPPD. Several studies regarding risk indicators of peri-implant disease presented different outcomes with respect to gender [40, 41, 42, 43]. All data taken together, it is plausible that men are more frequently affected with peri-implant disease, which is associated with increased PiPPD [3].

### 4.5. Restoration Type

Patients, who received a fixed partial prosthesis (bridge restoration), showed a higher increase in PiPPD compared to implant-supported single crowns (Table 2). This is supported by other research which showed more marginal bone loss around implant-supported fixed partial dentures compared to single implant-supported crowns [44]. Inadequate access to peri-implant hygiene frequently resulted in more peri-implant inflammation and marginal bone loss overtime [45]. Plaque has been identified as significant risk indicators for secondary implant failure due to peri-implantitis [46]. Proper accessibility to peri-implant hygiene should therefore be carefully considered during planning of implant restoration, and patients properly motivated into maintenance care. Although no plaque scores were recorded for this retrospective analysis one could speculate that fixed partial dentures may be more difficult to clean. However, also contradictory results have been shown where splinting implant-supported restorations may result in reduced stress on peri-implant bone and in doing so reduce the risk of marginal bone loss [47]. Unsplinted restorations resulted in significantly lower stress levels at the implant neck compared to splinted restorations. However, higher strain values were observed on the strain gauges located at the restoration margins of the single crowns, as compared to splinted restorations. This increased strain could potentially lead to restorative complications [48].

### 4.6. Emergence Angle

The emergence angle was found not to be a predictor of increased PiPPD at follow-up (Table 4) and neither any differences were found between the <30° and ≥30° groups (Table 2). The line of thoughts from the existing literature state that a restoration contour exceeding 30° is a risk indicator for peri-implant disease [17, 18]. Also our earlier work found a significant relationship between the emergence angle and the extent of change in the marginal bone level between the second stage surgery and the baseline assessment [22]. Therefore, it appears important to critically consider the restoration contour before implant placement to reduce the chances of peri-implant disease [22]. This is certainly the case if bone-level platform-switched implants are used [49]. Implants should, therefore, be placed at a sufficient depth. Limiting the depth of implant placement significantly restricts the available prosthetic options and hinders the restoration's ability to emerge from the implant platform to the desired contour. Consequently, shallow placement will lead to a sharper angle of the contour [50]. The absence of a significant association between the emergence angle and peri-implant health in our study may be explained by differences in study design, sample size, follow-up duration, or the influence of confounding variables such as oral hygiene practices, prosthetic design, and peri-implant maintenance protocols. It is also possible that the clinical impact of emergence angle is less pronounced in well-maintained patients or when other risk factors are well controlled. Further longitudinal studies are warranted to clarify the role of emergence profile in peri-implant disease development.

### 4.7. Periodontal Status

The patients included in this retrospective analysis were either periodontally healthy or under periodontal maintenance. Analysis revealed no difference with respect to PiPPD between patients with pockets ≥5 mm or <5 mm around their natural teeth. A longer-term study with a follow-up of 6–8 years did, however, show that partially edentulous patients with the history of severe periodontitis expressed higher probability of peri-implantitis [51]. One could speculate that the observed difference is due to the lack of regular supportive periodontal care. However, a retrospective study conducted with 18–23 years follow-up found no significant association between regular maintenance care and the prevalence of peri-implantitis nor with a history of periodontitis and peri-implantitis [52]. Altogether it is important to note that observational studies like these do not establish cause–effect relationships, which could help explain their finding.

### 4.8. Limitations

Although the current findings indicate relatively stable peri-implant health conditions, it is essential to acknowledge several potential limitations. First, the retrospective nature of this study introduces certain constraints. Being a practice-based study, data were collected “a priori” for clinical purposes, which may result in incomplete information on confounding factors. Moreover, the relatively low number of smokers (18%; Table 1) made it impractical to conduct a comprehensive analysis of smoking status in relation to peri-implant health.

Additionally, practice-based studies generally yield less robust data compared to those collected in prospective research designs, as the primary focus was not on analyzing outcomes [53]. Nonetheless, practice-based studies do provide valuable insights from real-life situations, which can offer a better understanding of the scenarios encountered in general dental practice.

A second potential limitation may be the low adherence of patients to the maintenance visits. Only one-third of the patients adhered to the annual maintenance visits. A similar analysis regarding peri-implant tissue health encountered the same problem of adherence [39]. Also a systematic review concluded unsatisfactory compliance of patients to peri-implant maintenance therapy [54]. These data show the degree in difficulty of motivating patients to adhere to regular maintenance care. The limited compliance rate (33%) raises the possibility of attrition bias, as participants who failed to attend follow-up visits might have experienced less favorable outcomes. As a result, the reported clinical effects may not fully reflect the intervention's effectiveness across the entire study population. Peri-implant maintenance compliance is proven to be essential in preventing peri-implant disease and patients, who attended annual maintenance visits, also demonstrated better clinical conditions [55, 56].

A third limitation is that descriptive statistics show that most of the implants (70%) were placed in the maxilla and that only nine implants (9%) served as a replacement for anterior teeth (Table 1). Anterior teeth are the least frequently extracted teeth, explaining the skewed distribution [57]. An equal distribution would be more favorable to assess the relationship between implant position and peri-implant health more adequately.

Finally, a limitation may be the relatively few patients (Table 1) that are included in this study. A small sample size may result in an underpowered study making it more difficult to detect statistical significance [58].

### 4.9. Future Research

In terms of future research, it would be useful to further examine the possible association between gender and peri-implant health. Besides, the literature is so far not decisive regarding the association between marginal bone loss and increased PiPPD to restoration type (splinting vs. nonsplinting). Whether this association exists, is an intriguing question for future studies.

As the buccal keratinized mucosa around implants is an important factor to maintain the peri-implant health, and in case of guided bone regeneration procedure the advance of the flap reduces the buccal keratinized mucosa, future studies should provide information on this aspect [59].

Further, it would be interesting to compare the clinical conditions of patients participating in a maintenance program with those that did not. Also, a longer follow-up period would be of interest.

## 5. Conclusion

Despite the limitations of this retrospective analysis, the authors conclude that the peri-implant conditions remained fairly stable after the follow-up period. Finally, considering the number of patients that had to be excluded in general more effort needs to be made to improve the adherence of patients to peri-implant maintenance care.

## Figures and Tables

**Table 1:** Patient and implant demographics.

Age, mean (SD)	57.3 (9.6)
Age range in years	33–74
Number of male patients	26 (38%)
Number of female patients	42 (62%)
Follow-up duration in months, mean (SD)	47 (3.6)
Range in months of follow-up duration	42–54
Number of patients with the presence of ≥5 mm pockets	44 (65%)
Number of patients smoking	12 (18%)
Number of implants placed in the maxilla	76 (70%)
Number of implants placed in the mandibula	32 (30%)
Number of implants placed in the front region	9 (8.3%)
Number of implants placed in premolar region	51 (47.2%)
Number of implants placed in molar region	48 (44.4%)
Number of implant-supported single crown	72 (66.7%)
Number of implant-supported fixed partial denture (bridge)	36 (33.3%)
Number of 3.3-mm implants	52 (48%)
8-mm	7
10-mm	29
12-mm	16
Number of 4.1-mm implants	56 (52%)
8-mm	9
10-mm	34
12-mm	13
Emergence angle at the mesial site in degrees, mean (SD)	31.5 (10.5)
Emergence angle at the distal site in degrees, mean (SD)	32 (10.1)

**Table 2:** Clinical parameters at implant level of overall mean (SD) peri-implant pocket probing depth (PiPPD) in millimeters and peri-implant bleeding on probing (PiBOP) at baseline and follow-up.

Categories	Baseline	Follow-up	Difference^b^	*p*-Value
PiBOP (*n* = 108)	31% (33)	48% (33)	17% (39)	**<0.001** ^a^
PiPPD (*n* = 108)	3.16 (0.79)	3.48 (0.82)	0.32 (0.89)	**<0.001** ^a^
Implant diameter				
3.3 mm (*n* = 52)	3.07 (0.67)	3.54 (0.94)	0.48 (1.0)	**0.001** ^a^
4.1 mm (*n* = 56)	3.24 (0.89)	3.41 (0.69)	0.17 (0.75)	0.097^a^
				0.074^b^
Implant location				
Anterior (*n* = 9)	3.19 (0.78)	3.36 (0.96)	0.17 (0.65)	0.464^a^
Posterior (*n* = 99)	3.15 (0.80)	3.48 (0.81)	0.33 (0.91)	**<0.001** ^a^
				0.599^b^
Jaw distribution				
Maxilla (*n* = 76)	3.28 (0.84)	3.60 (0.75)	0.32 (0.84)	**0.001** ^a^
Mandibula (*n* = 32)	2.87 (0.59)	3.17 (0.91)	0.30 (1.02)	0.102^a^
				0.926^b^
Gender				
Male (*n* = 42)	3.13 (0.65)	3.70 (0.93)	0.57 (1.03)	**<0.001** ^a^
Female (*n* = 66)	3.17 (0.87)	3.33 (0.71)	0.16 (0.75)	0.097^a^
				**0.017** ^b^
Smoking				
Yes (*n* = 23)	3.20 (1.0)	3.61 (0.89)	0.41 (1.19)	0.111^a^
No (*n* = 85)	3.15 (0.73)	3.44 (0.81)	0.29 (0.80)	**0.001** ^a^
				0.563^b^
Restoration				
Crown (*n* = 72)	3.30 (0.86)	3.45 (0.78)	0.15 (0.81)	0.116^a^
Fixed prothesis (bridge; *n* = 36)	2.88 (0.56)	3.52 (0.90)	0.65 (0.96)	**<0.001** ^a^
				**0.006** ^b^
Emergence angle				
≥30° (*n* = 67)	3.16 (0.82)	3.49 (0.81)	0.32 (0.89)	**0.004** ^a^
<30° (*n* = 41)	3.15 (0.76)	3.46 (0.85)	0.30 (0.91)	**0.038** ^a^
				0.912^b^

*Note:* Mean (SD) subanalysis by implant diameter, gender, smoking, restoration, and emergence angle. Bold values are statistically signficant.

^a^Paired-samples *T*-test, within-group comparison.

^b^Independent-samples *T*-test, between-group comparison.

**Table 3:** Clinical parameters at patient level of overall mean (SD) peri-implant pocket probing depth (PiPPD) in millimeters and peri-implant bleeding on probing (PiBOP) at baseline and follow-up.

Categories	Baseline	Follow-up	Difference^b^	*p*-Value^a^
PiBOP (*n* = 68)	31.2% (32)	51.0% (29)	19.8% (33)	**<0.001** ^a^
PiPPD (*n* = 68)	3.07 (0.66)	3.45 (0.74)	0.38 (0.75)	**<0.001** ^a^
Gender				
Male (*n* = 26)	3.04 (0.57)	3.65 (0.73)	0.62 (0.81)	**<0.001** ^a^
Female (*n* = 42)	3.09 (0.72)	3.32 (0.72)	0.23 (0.68)	**0.031** ^a^
				**0.039** ^b^
Smoking				
Yes (*n* = 12)	3.03 (0.55)	3.52 (0.78)	0.49 (0.94)	0.095^a^
No (*n* = 56)	3.08 (0.69)	3.43 (0.73)	0.36 (0.71)	**<0.001** ^a^
				0.536^b^
Presence of pockets ≥5 mm				
Yes (*n* = 44)	3.25 (0.71)	3.53 (0.71)	0.28 (0.70)	**0.013** ^a^
No (*n* = 24)	2.73 (0.39)	3.30 (0.78)	0.58 (0.80)	**0.002** ^a^
				0.118^b^

*Note:* Mean (SD) subanalysis by gender, smoking, and PPD. Bold values are statistically signficant.

^a^Paired-samples *T*-test, within-group comparison.

^b^Independent-samples *T*-test, between-group comparison.

**Table 4:** Multivariate linear regression analysis for predicting peri-implant pocket probing depth (PiPPD) at follow-up.

Variable	Unstand. Coef. ß	95% CI for ß	Stand. Coef. ß	*p*-Value
Baseline PiPPD	0.42	[0.23; 0.47]	0.40	**<0.001**
Mean emergence angle	0.01	[−0.01; 0.029]	0.12	0.187
Age at implant placement	0.006	[−0.01; 0.022]	0.07	0.437
Smoking (ref.: not smoking)	0.12	[−0.23; 0.47]	0.06	0.510
Wide-diameter (ref.: narrow-diameter)	−0.12	[−0.42; 0.18]	−0.07	0.431
Male (ref.: female)	0.38	[0.07; 0.69]	0.22	**0.017**

*Note:* Ref., reference category. *R*^2^ = 0.23; *F* = 5.1 (*p*=<0.001). Bold values are statistically signficant.

Abbreviations: Stand. Coef., standardized coefficients; Unstand. Coef., unstandardized coefficients.

## Data Availability

Supporting information, such as raw data, is available from the corresponding author upon reasonable request. We used the STROBE checklist as a reporting guideline.
